# Anti-Tumor Effects of Novel 5-*O*-Acyl Plumbagins Based on the Inhibition of Mammalian DNA Replicative Polymerase Activity

**DOI:** 10.1371/journal.pone.0088736

**Published:** 2014-02-10

**Authors:** Moe Kawamura, Isoko Kuriyama, Sayako Maruo, Kouji Kuramochi, Kazunori Tsubaki, Hiromi Yoshida, Yoshiyuki Mizushina

**Affiliations:** 1 Graduate School of Life and Environmental Sciences, Kyoto Prefectural University, Kyoto, Kyoto, Japan; 2 Laboratory of Food & Nutritional Sciences, Faculty of Nutrition, Kobe Gakuin University, Kobe, Hyogo, Japan; 3 Cooperative Research Center of Life Sciences, Kobe Gakuin University, Kobe, Hyogo, Japan; Kobe University, Japan

## Abstract

We previously found that vitamin K_3_ (menadione, 2-methyl-1,4-naphthoquinone) inhibits the activity of human mitochondrial DNA polymerase γ (pol γ). In this study, we focused on plumbagin (5-hydroxy-2-methyl-1,4-naphthoquinone), and chemically synthesized novel plumbagins conjugated with C2:0 to C22:6 fatty acids (5-*O*-acyl plumbagins). These chemically modified plumbagins enhanced mammalian pol inhibition and their cytotoxic activity. Plumbagin conjugated with chains consisting of more than C18-unsaturated fatty acids strongly inhibited the activities of calf pol α and human pol γ. Plumbagin conjugated with oleic acid (C18:1-acyl plumbagin) showed the strongest suppression of human colon carcinoma (HCT116) cell proliferation among the ten synthesized 5-*O*-acyl plumbagins. The inhibitory activity on pol α, a DNA replicative pol, by these compounds showed high correlation with their cancer cell proliferation suppressive activity. C18:1-Acyl plumbagin selectively inhibited the activities of mammalian pol species, but did not influence the activities of other pols and DNA metabolic enzymes tested. This compound inhibited the proliferation of various human cancer cell lines, and was the cytotoxic inhibitor showing strongest inhibition towards HT-29 colon cancer cells (LD_50_ = 2.9 µM) among the nine cell lines tested. In an *in vivo* anti-tumor assay conducted on nude mice bearing solid tumors of HT-29 cells, C18:1-acyl plumbagin was shown to be a promising tumor suppressor. These data indicate that novel 5-*O*-acyl plumbagins act as anti-cancer agents based on mammalian DNA replicative pol α inhibition. Moreover, the results suggest that acylation of plumbagin is an effective chemical modification to improve the anti-cancer activity of vitamin K_3_ derivatives, such as plumbagin.

## Introduction

Cancer is a major global public health problem. Epidemiological and animal studies have indicated that chemopreventive natural products are associated with a reduced risk of cancer development [1], [2]. Furthermore, selective inhibitors of DNA polymerases (pols) are considered potentially useful anti-cancer, anti-viral, anti-parasitic, and anti-pregnancy agents because some are known to suppress human cancer and normal cell proliferation, and are cytotoxic [3], [4].

Pol (DNA-dependent DNA polymerase, E.C. 2.7.7.7) catalyzes deoxyribonucleotide addition to the 3′-hydroxyl terminus of primed double-stranded DNA (dsDNA) molecules [5]. The human genome encodes at least 15 DNA pols, which function in cellular DNA synthesis [6], [7]. Eukaryotic cells contain three replicative pols (α, δ, and ε), one mitochondrial pol (γ), and at least 11 non-replicative pols [β, ζ, η, θ, ι, κ, λ, μ, ν, terminal deoxynucleotidyl transferase (TdT), and REV1] [8], [9]. Pols have a highly conserved structure, with their overall catalytic subunits showing little variation among species; conserved enzyme structures are usually preserved over time as they perform important cellular functions that confer evolutionary advantages. Based on sequence homology, eukaryotic pols can be divided into four main families, termed A, B, X, and Y [8]. Family A includes mitochondrial pol γ as well as pols θ and ν; family B includes the three replicative pols α, δ, and ε and also pol ζ; family X comprises pols β, λ, and μ as well as TdT; and family Y includes pols η, ι, and κ in addition to REV1 [9]. We have studied selective inhibitors of each pol derived from natural products including food materials and nutrients for more than 18 years [10], [11]. We have found that vitamin K_3_, but not K_1_ or K_2_, is a potent inhibitor of human pol γ [12], [13].

**Figure 1:**
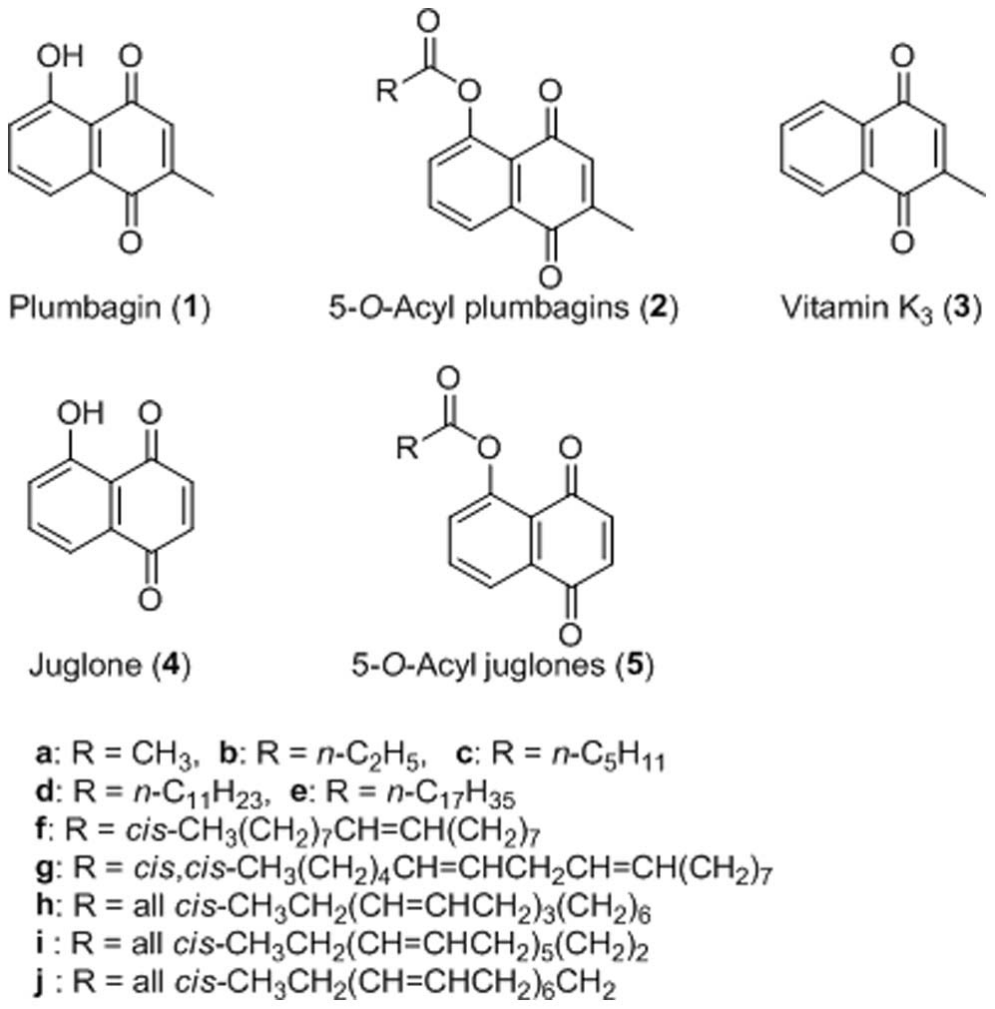
Structure of plumbagin (1), 5-*O*-acyl plumbagins (2), vitamin K_3_ (3), juglone (4), and 5-*O*-acyl juglones (5). “R” represents a saturated or unsaturated alkyl group in 5-*O*-acyl plumbagins (**2**) and 5-*O*-acyl juglones (**5**).

Vitamin K_3_ (menadione, 2-methyl-1,4-naphthoquinone, **3** of Fig. 1) is a fat-soluble compound that contains quinone as its principal chemical feature. Quinones are a class of organic compounds that are derived from aromatic compounds via the exchange of an even number of –CH = groups for –C( = O)– groups and any necessary rearrangement of double bonds, resulting in a fully conjugated cyclic dione structure. The toxicological properties of quinones, which act as alkylating agents, have also been examined. For example, quinones are known to interact with flavoproteins to generate reactive oxygen species (ROS) that can induce biological injury [14]–[17]. In this study, we focused on 5-hydroxy-2-methyl-1,4-naphthoquinone (plumbagin, **1** of Fig. 1), which has the common naphthoquinone skeleton and a hydroxyl group and a methyl group at the C-5 and C-2 positions, respectively. Plumbagin (**1**) is found in the plants of the Plumbaginaceae, Droseraceae, Ancestrocladaceae, and Dioncophyllaceae families. The chief source of plumbagin (**1**) is the root of *Plumbago zeylanica* L. (also known as “Chitrak”). Plumbagin (**1**) has been shown to exert anti-carcinogenic, anti-atherosclerotic, and anti-microbial effects [18]–[21]. The root of *P. zeylanica* L. has been used in Indian medicine for approximately 2,750 years and its components possess anti-atherogenic, cardiotonic, hepatoprotective, and neuroprotective properties [19]. Plumbagin (**1**) has potent anti-proliferative and apoptotic activities in various types of human cancers, and its mechanism of cytotoxicity is by inhibition of a PI-5 kinase for ROS generation [22].

We previously found that a vitamin K_3_ (**3**) derivative, juglone (5-hydroxy-1,4-naphthoquinone, **4** of Fig. 1), conjugated with fatty acids such as 5-*O*-acyl juglones (**5a–j** of Fig. 1) were stronger pol inhibitors than juglone alone (**4**) [23], therefore, ten 5-*O*-acylated derivatives of plumbagin (**2a–j** of Fig. 1) were chemically synthesized from plumbagin (**1**) and fatty acids to compare with 5-*O*-acyl juglones (**5a–j**). In this study, we first investigated the stability of 5-*O*-acyl plumbagins (**2**) and 5-*O*-acyl juglones (**5**). We also assayed the inhibitory effects of ten 5-*O*-acyl plumbagins (**2a–j**) on mammalian pol activity, cytotoxicity in human cancer cell lines and *in vivo* anti-tumor activity compared with plumbagin (**1**) and 5-*O*-acyl juglones (**5**). The relationship between the pol inhibitory and anti-tumor effects of vitamin K_3_-based acylated derivatives is discussed.

## Materials and Methods

### Materials

A chemically synthesized DNA template, poly(dA), was purchased from Sigma-Aldrich Inc. and a customized oligo(dT)_18_ DNA primer was produced by Sigma-Aldrich Japan K.K. (Hokkaido, Japan). Radioactive nucleotide [^3^H]-labeled 2′-deoxythymidine-5′-triphosphate (dTTP; 43 Ci/mmol) was obtained from Moravek Biochemicals Inc. (Brea, CA, USA). All other reagents were of analytical grade from Nacalai Tesque Inc. (Kyoto, Japan).

### Enzymes

Pol α was purified from calf thymus by immunoaffinity column chromatography as described by Tamai et al. [24]. Recombinant rat pol β was purified from *Escherichia coli* JMpβ5 as described by Date et al. [25]. The human pol γ catalytic gene was cloned into pFastBac. Histidine-tagged enzyme was expressed using the BACTO-BAC HT Baculovirus Expression System according to the manufacturer's instructions (Life Technologies, Frederick, MD, USA) and purified using ProBound resin (Invitrogen Japan, Tokyo Japan) [26]. Human pols δ and ε were purified by nuclear fractionation of human peripheral blood cancer cells (Molt-4) using the second subunit of pol δ and ε-conjugated affinity column chromatography, respectively [27]. A truncated form of human pol η (residues 1–511) tagged with His_6_ at its C-terminal was expressed in *E. coli* cells and purified as described by Kusumoto et al. [28]. A recombinant mouse pol ι tagged with His_6_ at its C-terminal was expressed and purified by Ni–NTA column chromatography [29]. A truncated form of pol κ (residues 1–560) with 6× His-tags attached at the C-terminus was overexpressed in *E. coli* and purified as described by Ohashi et al. [30]. Recombinant human His-pol λ was overexpressed and purified according to a method described by Shimazaki et al. [31]. Recombinant human His-pol μ was overexpressed in *E. coli* BL21 and purified using Glutathione Sepharose 4B (GE Healthcare Bio-Science Corp., Piscataway, NJ, USA) column chromatography according to the method for pol λ preparation by Shimazaki et al. [31]. Pol α from a higher plant, cauliflower, was purified from the inflorescence structure according to the methods outlined by Sakaguchi et al. [32]. Recombinant rice (*Oryza sativa* L. cv. Nipponbare) pol λ tagged with His_6_ at the C-terminus was expressed in *E. coli* and purified as described by Uchiyama et al. [33]. Calf TdT, *Taq* pol, T4 pol, T7 RNA polymerase, and T4 polynucleotide kinase were purchased from Takara Bio Inc. (Kyoto, Japan). The Klenow fragment of pol I from *E. coli* was purchased from Worthington Biochemical Corp. (Freehold, NJ, USA). Bovine pancreas deoxyribonuclease I was obtained from Stratagene Cloning Systems (La Jolla, CA, USA).

### Measurement of pol activity

The reaction mixtures for calf pol α, rat pol β, plant pol α, and prokaryotic pols have been described previously [34], [35]; those for pol γ as well as pols δ and ε were previously described by Umeda et al. [26] and Ogawa et al. [36], respectively. Those for pols η, ι, and κ were the same as for pol α, and those for pols λ and μ were the same as for pol β. For the pol reactions, poly(dA)/oligo(dT)_18_ (A/T, 2/1) and dTTP were used as the DNA template-primer substrate and nucleotide (dNTP; 2′-deoxynucleoside-5′-triphosphate) substrate, respectively. For the TdT reactions, oligo(dT)_18_ (3′-OH) and dTTP were used as the DNA primer substrate and nucleotide substrate, respectively.

The chemically synthesized 5-*O*-acyl plumbagins (**2a–j**) were dissolved in distilled dimethyl sulfoxide (DMSO) to various concentrations and sonicated for 30 s. Subsequently, 4 µL aliquots were mixed with 16 µL of each enzyme (0.05 units) in 50 mM Tris-HCl at pH 7.5, containing 1 mM dithiothreitol, 50% glycerol (by vol), and 0.1 mM ethylenediaminetetraacetic acid and held at 0°C for 10 min. These inhibitor-enzyme mixtures in 8 µL volumes were next added to 16 µL of enzyme standard reaction mixture and incubated at 37°C for 60 min, except for *Taq* pol, which was incubated at 74°C for 60 min. Activity without inhibitor was considered 100%, and the relative activity was determined for each inhibitor concentration. One unit of pol activity was defined as the amount of each enzyme that catalyzed the incorporation of 1 nmol dTTP into synthetic DNA template primers in 60 min, at 37°C, and under standard reaction conditions [34], [35].

### Other enzyme assays

The activities of calf primase of pol α, T7 RNA polymerase, mouse inosine-5′-triphosphate (IMP) dehydrogenase (type II), T4 polynucleotide kinase, and bovine deoxyribonuclease I were measured in standard assays according to the manufacturer's specifications, as described by Tamiya-Koizumi et al. [37], Nakayama and Saneyoshi [38], Mizushina et al. [39], Soltis et al. [40], and Lu and Sakaguchi [41], respectively.

### Thermal transition of DNA

Thermal transition profiles of dsDNA to single-stranded DNA with or without test compound were obtained with a spectrophotometer (UV-2500; Shimadzu Corp., Kyoto, Japan) equipped with a thermoelectric cell holder according to previous methods [42]. Calf thymus DNA (6 µg/mL) was dissolved in 0.1 M sodium phosphate buffer (pH 7.0) containing 1% DMSO. The solution temperature was equilibrated to 75°C for 10 min, and then increased by 1°C at 2-min intervals for each measurement point. Any change in the absorbance (260 nm) of the compound itself at each temperature point was automatically subtracted from that of DNA plus the compound in the spectrophotometer.

### Cell culture and measurement of cancer cell viability

The following human cancer cell lines were obtained from the American Type Culture Collection (Manassas, VA, USA): lung (A549), prostate (DU145 and PC3), colon (HCT116 and HT-29), cervix (HeLa), hepatocellular liver (HepG2), breast (MCF-7), and pancreatic cancer (PANC-1). These cells were cultured in RPMI 1640 medium supplemented with 10% fetal bovine serum, penicillin (100 units/mL), streptomycin (100 µg/mL), and 1.6 mg/mL NaHCO_3_ at 37°C in a humid atmosphere of 5% CO_2_/95% air. For the cell viability assay, cells were seeded at 1×10^3^ cells/well in a 96-well microplate with various concentrations of the test compounds, and incubated for 48 h. MTT (3-(4,5-dimethylthiazol-2-yl)-2,5-diphenyl tetrazolium bromide) solution was added to a final concentration 0.6 mg/mL in Milli-Q purified water for 2 h [43], after which time the medium was discarded and the cells lysed in DMSO. A_540_ was then measured in a microplate reader (Model 680, Bio-Rad Laboratories, Hercules, CA, USA).

### *In vivo* assessment of anti-tumor assay

Male BALB/c nu/nu mice at 5 week of age (18 g) were purchased from Japan SLC, Inc. (Shizuoka, Japan). Mice receiving standard laboratory chow and water *ad libitum* were acclimatized for 1 week before inoculation with cancer cells. For *in vivo* experiments, human colon HT-29 cells (1×10^7^ cells/mouse) were subcutaneously injected into nude mice. Mice bearing solid tumors that had grown to approximately 100 mm^3^ in volume (tumor volume = length×width×height) at 12 days after implantation were used for the assessment of anti-tumor effects. They were divided randomly into five groups (n = 6/group). One of the five groups was a control group injected with 0.1 mL of phosphate buffered saline (PBS) alone, and other groups were injected with vitamin K_3_ (**3**), juglone (**4**), C18:1-acyl juglone (**5f**), and C18:1-acyl plumbagin (**2f**) dissolved in PBS at a dose of 5 mg/kg. The above administrations all took place between days 12–39 subsequent to implantation. All mice were injected subcutaneously 14 times at 1-day intervals with the compound and PBS alone (control). Tumor growth was measured at 1-day intervals for 40 days after implantation. At the end of the *in vivo* anti-tumor assay, some mice treated with the test compounds and PBS were independently examined to observe major organs such as lung, heart, spleen, stomach, liver, pancreas, kidney, intestine, and brain.

This animal study was approved by the Institutional Animal Care and Use Committee of Kobe Gakuin University, and was performed according to the guidelines outlined in the Care and Use of Laboratory Animals of Kobe Gakuin University. The animals were anesthetized with pentobarbital before undergoing cervical dislocation. The mice that had been bred in-house with free access to food and water were used for all experiments. All of the mice were maintained under a 12-h light/dark cycle and housed at a room temperature of 25°C.

### Analytical instruments for synthetic 5-O-acyl plumbagins (2a–j)

Melting points, determined on a Yanaco Micro Melting Point apparatus, are uncorrected. NMR spectra were recorded on a Bruker spectrometer (Avance 400). Chemical shifts are expressed in δ (ppm) relative to Me_4_Si or the residual solvent resonance, and coupling constants (*J*) are expressed in Hz. The following abbreviations are used for spin multiplicity: s = singlet, d = doublet, t = triplet, q = quartet, quin = quintet, m = multiplet, br = broad. Infrared spectra (IR) were recorded on a HORIBA FT-720, using NaCl (neat) or KBr pellets (solid) and are reported in wavenumbers (cm^−1^). High resolution mass spectra (HRMS) were obtained on a JEOL mass spectrometer (JMS-700 MStaion) using fast atom bombardment (FAB), or a Fourier transformation-ion cyclotron resonance-mass spectrometer, Bruker solariX (FT-ICR-MS) by using electrospray ionization (ESI) and laser desorption ionization (LDI) techniques. Analytical thin-layer chromatography (TLC) was performed on Silica Gel 60 F_254_ plates (Merck). Flash chromatography was carried out on SiliaFlash F60 (Silicycle).

### Synthesis and characterization of 5-O-acetoxy-2-methyl-1,4-naphthoquinone (C2:0-Acyl plumbagin, 2a)

Acetic anhydride (0.5 mL) was added to a solution of plumbagin (**1**) (107 mg, 0.57 mmol) in pyridine (1.0 mL), and the mixture was stirred at room temperature for 4 h. After the solvent was removed, the residue was purified by silica gel chromatography (EtOAc/hexanes 1∶3, v/v) to yield **2a** (118 mg, 90%) as a yellow solid [44]. Mp 118–124°C; IR (KBr) 3049, 2987, 2966, 2927, 1761, 1662, 1593, 1431, 1375, 1365, 1271, 1203, 1024, 910, 785 cm^−1^; ^1^H NMR (400 MHz, CDCl_3_) δ 8.06 (dd, *J* = 8.0 Hz, 0.8 Hz, 1H), 7.73 (t, *J* = 8.0 Hz, 1H), 7.36 (dd, *J* = 8.0 Hz, 0.8 Hz, 1H), 6.71 (q, *J* = 1.2 Hz, 1H), 2.45 (s, 3H), 2.17 (d, *J* = 1.2 Hz, 3H); ^13^C NMR (100 MHz, CDCl_3_) δ 184.8, 183.6, 169.4, 149.2, 146.9, 136.8, 134.4, 133.8, 129.4, 125.1, 123.4, 21.1, 16.0; HRMS (FAB) calcd for C_13_H_10_O_4_Na ([M+Na]^+^) 253.0477, found 253.0482

### General procedure for the preparation of 5-O-acyl plumbagins (2b–f) using MNBA

Et_3_N (2.5–2.6 equiv.), 2-methyl-6-nitrobenzoic anhydride (MNBA) (1.5 equiv.) and *N,N*-dimethyl-4-aminopyridine (DMAP) (0.1 equiv.) were added to a solution of plumbagin (**1**) (1.0 equiv.) and carboxylic acid (1.5 equiv.) in CH_2_Cl_2_ at room temperature. The mixture was stirred at room temperature under a N_2_ atmosphere until no further TLC changes were observed. The reaction was quenched by the addition of H_2_O, and the mixture was extracted with CHCl_3_. The combined extracts were washed with brine, dried over Na_2_SO_4_, and concentrated. The residue was purified by silica gel chromatography using EtOAc/hexanes as eluent.

### Synthesis and characterization of 5-O-propanolyloxy-2-methyl-1,4-naphthoquinone (C3:0-Acyl plumbagin, 2b)

Following the general procedure, the reaction of plumbagin (**1**) (52 mg, 0.28 mmol) with propionic acid (30 µL, 0.41 mmol) using MNBA (143 mg, 0.41 mmol), Et_3_N (96 µL, 0.69 mmol), and DMAP (3.4 mg, 0.03 mmol) for 2 h gave the crude product. The crude product was purified by silica gel column chromatography (EtOAc/hexanes = 1/5, v/v) to give **2b** (60 mg, 89%) as a yellow solid [44]. Mp = 108–112°C; IR (KBr) 3076, 3043, 2981, 2937, 1766, 1662, 1630, 1591, 1358, 1265, 1194, 1132, 1080, 1024, 904, 881, 781 cm^−1^; ^1^H NMR (400 MHz, CDCl_3_) δ 8.06 (dd, *J* = 8.0 Hz, 1.2 Hz, 1H), 7.73 (t, *J* = 8.0 Hz, 1H), 7.36 (dd, *J* = 8.0 Hz, 1.2 Hz, 1H), 6.71 (q, *J* = 1.2 Hz, 1H), 2.77 (q, *J* = 7.6 Hz, 2H), 2.16 (d, *J* = 1.2 Hz, 3H), 1.32 (t, *J* = 7.6 Hz, 3H); ^13^C NMR (100 MHz, CDCl_3_) δ 184.8, 183.6, 172.8, 149.3, 146.8, 136.9, 134.4, 133.8, 129.4, 125.0, 123.5, 27.6, 16.0, 8.7; HRMS (FAB) calcd for C_14_H_12_O_4_Na ([M+Na]^+^) 267.0633, found 267.0639.

### Synthesis and characterization of 5-O-hexanoyloxy-2-methyl-1,4-naphthoquinone (C6:0-Acyl plumbagin, 2c)

Following the general procedure, the reaction of plumbagin (**1**) (100 mg, 0.53 mmol) with caproic acid (100 µL, 0.80 mmol) using MNBA (274 mg, 0.80 mmol), Et_3_N (185 µL, 1.33 mmol), and DMAP (6.7 mg, 0.05 mmol) for 2 h gave the crude product. The crude product was purified by silica gel column chromatography (EtOAc/hexanes = 1/6, v/v) to give **2c** (102 mg, 68%) as a yellow solid. Mp = 45–47°C; IR (KBr) 3086, 2958, 2929, 2871, 1766, 1664, 1631, 1595, 1454, 1362, 1267, 1234, 1138, 1105, 912, 891, 791 cm^−1^; ^1^H NMR (400 MHz, CDCl_3_) δ 7.99 (dd, *J* = 8.0 Hz, 1.2 Hz, 1H), 7.65 (t, *J* = 8.0 Hz, 1H), 7.28 (dd, *J* = 8.0 Hz, 1.2 Hz, 1H), 6.64 (q, *J* = 1.2 Hz, 1H), 2.66 (t, *J* = 7.6 Hz, 2H), 2.09 (d, *J* = 1.2 Hz, 3H), 1.75 (quin, *J* = 7.6 Hz, 2H), 1.41-1.28 (m, 4H), 0.87 (t, *J* = 7.6 Hz, 3H); ^13^C NMR (100 MHz, CDCl_3_) δ 184.9, 183.6, 172.2, 149.4, 146.8, 136.9, 134.4, 133.8, 129.5, 125.0, 123.6, 34.2, 31.3, 24.1, 22.4, 16.1, 14.0; HRMS (FAB) calcd for C_17_H_18_O_4_Na ([M+Na]^+^) 309.1103, found 309.1099.

### Synthesis and characterization of 5-O-dodecanoyloxy-2-methyl-1,4-naphthoquinone (C12:0-Acyl plumbagin, 2d)

Following the general procedure, the reaction of plumbagin (**1**) (53 mg, 0.28 mmol) with lauric acid (85 mg, 0.43 mmol) using MNBA (147 mg, 0.43 mmol), Et_3_N (100 µL, 0.72 mmol), and DMAP (3.5 mg, 0.03 mmol) for 19 h gave the crude product. The crude product was purified by silica gel column chromatography (EtOAc/hexanes = 1/9, v/v) to give **2d** (90 mg, 86%) as a yellow crystal; Mp 41–42°C; IR (KBr) 2922, 2850, 1755, 1654, 1632, 1593, 1464, 1358, 1271, 1230, 1188, 1144, 939, 918, 893, 787 cm^−1^; ^1^H NMR (400 MHz, CDCl_3_) δ 8.06 (dd, *J* = 8.0 Hz, 1.2 Hz, 1H), 7.73 (t, *J* = 8.0 Hz, 1H), 7.35 (dd, *J* = 8.0 Hz, 1.2 Hz, 1H), 6.71 (q, *J* = 1.2 Hz, 1H), 2.73 (t, *J* = 7.6 Hz, 2H), 2.16 (d, *J* = 1.2 Hz, 3H), 1.81 (quin, *J* = 7.6 Hz, 2H), 1.38-1.27 (brm, 16H), 0.88 (t, *J* = 7.6 Hz, 3H); ^13^C NMR (100 MHz, CDCl_3_) δ 184.9, 183.6, 172.2, 149.4, 146.8, 136.9, 134.4, 133.8, 129.5, 125.0, 123.6, 34.2, 31.9, 29.6 (2C), 29.5, 29.3, 29.3, 29.2, 24.4, 22.7, 16.1, 14.1; HRMS (ESI-LDI) calcd for C_23_H_30_O_4_Na ([M+Na]^+^) 393.2036, found 393.2032.

### Synthesis and characterization of 5-O-octadecanoyloxy-2-methyl-1,4-naphthoquinone (C18:0-Acyl plumbagin, 2e)

Following the general procedure, the reaction of plumbagin (**1**) (102 mg, 0.54 mmol) with stearic acid (231 mg, 0.81 mmol) using MNBA (280 mg, 0.81 mmol), Et_3_N (189 µL, 1.36 mmol), and DMAP (6.6 mg, 0.05 mmol) for 19 h gave the crude product. The crude product was purified by silica gel column chromatography (EtOAc/hexanes = 1/10, v/v) to give **2e** (139 mg, 56%) as a yellow solid. Mp = 67–70°C; IR (KBr) 2922, 2850, 1759, 1660, 1593, 1541, 1514, 1471, 1273, 1142, 1105, 893, 789 cm^−1^; ^1^H NMR (400 MHz, CDCl_3_) δ 7.99 (dd, *J* = 8.0 Hz, 1.2 Hz, 1H), 7.65 (t, *J* = 8.0 Hz, 1H), 7.27 (dd, *J* = 8.0 Hz, 1.2 Hz, 1H), 6.63 (q, *J* = 1.2 Hz, 1H), 2.66 (t, *J* = 7.6 Hz, 2H), 2.09 (d, *J* = 1.2 Hz, 3H), 1.74 (quin, *J* = 7.6 Hz, 2H), 1.31-1.19 (brm, 28H), 0.81 (t, *J* = 7.6 Hz, 3H); ^13^C NMR (100 MHz, CDCl_3_) δ 184.9, 183,6, 172.2, 149.4, 146.8, 136.9, 134.4, 133.8, 129.5, 125.0, 123.6, 34.2, 31.9, 29.7 (5C), 29.6 (2C), 29.6, 29.5, 29.3, 29.2, 29.2, 24.4, 22.7, 16.0, 14.1; HRMS (FAB) calcd for C_29_H_42_O_4_ Na ([M+Na]^+^) 477.2981, found 447.2988.

### Synthesis and characterization of 5-O-oleoyl-2-methyl-1,4-naphthoquinone (C18:1-Acyl plumbagin, 2f)

Following the general procedure, the reaction of plumbagin (**1**) (100 mg, 0.53 mmol) with oleic acid (0.25 mL, 0.79 mmol) using MNBA (275 mg, 0.80 mmol), Et_3_N (186 µL, 1.33 mmol), and DMAP (6.5 mg, 0.05 mmol) for 24 h gave the crude product. The crude product was purified by silica gel column chromatography (EtOAc/hexanes = 1/20, v/v) to give **2f** (166 mg, 69%) as yellow oil. IR (neat) 3005, 2925, 2854, 1770, 1664, 1595, 1462, 1356, 1271, 1190, 1107, 895, 783 cm^−1^; ^1^H NMR (400 MHz, CDCl_3_) δ 8.06 (dd, *J* = 8.0 Hz, 1.2 Hz, 1H), 7.72 (t, *J* = 8.0 Hz, 1H), 7.34 (dd, *J* = 8.0 Hz, 1.2 Hz, 1H), 6.70 (q, *J* = 1.2 Hz, 1H), 5.40-5.35 (m, 2H), 2.73 (t, *J* = 7.6 Hz, 2H), 2.16 (d, *J* = 1.2 Hz, 3H), 2.06-1.97 (brm, 4H), 1.81 (quin, *J* = 7.6 Hz, 2H), 1.47-1.27 (brm, 20H), 0.88 (t, *J* = 7.6 Hz, 3H); ^13^C NMR (100 MHz, CDCl_3_) δ 184.9, 183.6, 172.1, 149.3, 146.8, 136.9, 134.4, 133.8, 130.0, 129.7, 129.5, 125.0, 123.5, 34.2, 31.9, 29.8, 29.7, 29.6, 29.6, 29.5, 29.3, 29.2, 29.1, 27.2, 27.2, 24.4, 22.7, 16.1, 14.1; HRMS (FAB) calcd for C_29_H_40_O_4_Na ([M+Na]^+^) 475.2824, found 475.2821.

### General procedure for the preparation of acyl plumbagins (2g–j) via acyl chlorides

Oxalyl chloride (3.0–3.6 equiv.) was added to a solution of carboxylic acid (1 equiv.) in CH_2_Cl_2_ at 0°C. The mixture was stirred at room temperature for 3–4.5 h. The solvent was removed to yield crude acyl chloride. A solution of the acyl chloride (2.2–5.8 equiv.), plumbagin (**1**) (1 equiv.) and DMAP (0.1 equiv.) in pyridine was stirred at room temperature. The mixture was stirred at room temperature under a N_2_ atmosphere until no further TLC changes were observed. The reaction was quenched by the addition of H_2_O, and the mixture was extracted with CHCl_3_. The extracts were washed with brine, dried over Na_2_SO_4_, and concentrated. The residue was purified by silica gel chromatography using hexanes/ethyl acetate as eluent.

### Synthesis and characterization of 5-O-linoleoyloxy-2-methyl-1,4-naphthoquinone (C18:2-Acyl plumbagin, 2g)

Following the general procedure, linoleoyl chloride was prepared by treatment of linoleic acid (110 mg, 0.39 mmol) with oxalyl chloride (100 µL, 1.16 mmol) for 4 h. The reaction of plumbagin (**1**) (34 mg, 0.18 mmol) with the crude linoleoyl chloride using DMAP (2.2 mg, 0.02 mmol) in pyridine for 2 h gave the crude product. The crude product was purified by silica gel column chromatography (toluene/hexanes = 1/10, v/v) to give **2g** (31.9 mg, 39%) as yellow oil. IR (neat) 3008, 2927, 2854, 1768, 1664, 1595, 1462, 1358, 1271, 1190, 1009, 1026, 982, 895, 783 cm^−1^; ^1^H NMR (400 MHz, CDCl_3_) δ 8.06 (dd, *J* = 8.0 Hz, 1.2 Hz, 1H), 7.73 (t, *J* = 8.0 Hz, 1H), 7.35 (dd, *J* = 8.0 Hz, 1.2 Hz, 1H), 6.71 (q, *J* = 1.2 Hz, 1H), 5.42-5.33 (m, 4H), 2.78 (t, *J* = 7.6 Hz, 2H), 2.73 (t, *J* = 7.6 Hz, 2H), 2.16 (d, *J* = 1.2 Hz, 3H), 2.08-2.03 (m, 4H), 1.81 (quin, *J* = 7.6 Hz, 2H), 1.36-1.26 (m, 14H), 0.94-0.86 (m, 3H); ^13^C NMR (100 MHz, CDCl_3_) δ 184.9, 183.6, 172.2, 149.3, 146.8, 136.9, 134.4, 133.8, 130.2, 130.1, 129.5, 128.0, 127.9, 125.0, 123.5, 34.2, 32.5, 31.5, 29.6, 29.5, 29.3, 29.2, 29.1, 27.2, 25.6, 24.4, 22.6, 16.1, 14.1; HRMS (FAB) calcd for C_29_H_38_O_4_Na ([M+Na]^+^) 473.2668, found 473.2666.

### Synthesis and characterization of 5-O-linolenoyloxy-2-metyl-1,4-naphthoquinone (C18:3-Acyl plumbagin, 2h)

Following the general procedure, α-linolenoyl chloride was prepared by treatment of α-linolenic acid (200 mg, 0.72 mmol) with oxalyl chloride (200 µL, 2.33 mmol) for 4.5 h. The reaction of plumbagin (**1**) (25 mg, 0.13 mmol) with the crude α-linolenoyl chloride using DMAP (1.6 mg, 0.01 mmol) in pyridine for 18 h gave the crude product. The crude product was purified by silica gel column chromatography (EtOAc/hexanes = 1/20, v/v) to give **2h** (32 mg, 54%) as yellow oil; IR (neat) 3010, 2927, 2856, 1768, 1664, 1595, 1462, 1358, 1271, 1107, 974, 912, 783 cm^−1^; ^1^H NMR (400 MHz, CDCl_3_) δ 8.05 (dd, *J* = 7.6 Hz, 1.2 Hz, 1H), 7.71 (t, *J* = 7.6 Hz, 1H), 7.34 (dd, *J* = 7.6 Hz, 1.2 Hz, 1H), 6.70 (q, *J* = 1.2 Hz, 1H), 5.41-5.33 (m, 6H), 2.83-2.71 (m, 6H), 2.16 (d, *J* = 1.2 Hz, 3H), 2.12-2.00 (m, 4H), 1.81 (quin, 2H), 1.48-1.30 (brm, 8H), 0.98 (t, *J* = 7.6 Hz, 3H); ^13^C NMR (100 MHz, CDCl_3_) δ 184.9, 183.6, 172.2, 149.4, 146.8, 136.9, 134.4, 133.8, 132.0, 130.3, 130.0, 128.2 (2C), 127.7, 127.1, 125.0, 123.6, 34.2, 29.6, 29.2, 29.1, 27.2, 25.6, 25.5, 25.5, 24.4, 20.5, 16.1, 14.3; HRMS (FAB) calcd for C_29_H_37_O_4_ ([M+H]^+^) 449.2692, found 449.2694.

### Synthesis and characterization of 5-O-eicosapentaenoyloxy-2-methyl-1,4-naphthoquinone (C20:5-Acyl plumbagin, 2i)

Following the general procedure, eicosapentaenoyl chloride was prepared by treatment of eicosapentaenoic acid (194 mg, 0.64 mmol) with oxalyl chloride (200 µL, 2.33 mmol) for 3 h. The reaction of plumbagin (**1**) (20 mg, 0.10 mmol) with the crude eicosapentaenoyl chloride using DMAP (1.3 mg, 0.01 mmol) in pyridine for 4 h gave the crude product. The crude product was purified by silica gel column chromatography (EtOAc/hexanes = 1/20, v/v) to give **4i** (40 mg, 80%) as yellow oil. IR (neat) 3012, 2962, 1768, 1664, 1595, 1446, 1358, 1271, 1190, 1126, 897, 783, 717 cm^−1^; ^1^H NMR (400 MHz, CDCl_3_) δ 8.06 (dd, *J* = 7.6 Hz, 1.2 Hz, 1H), 7.72 (t, *J* = 7.6 Hz, 1H), 7.34 (dd, *J* = 7.6 Hz, 1.2 Hz, 1H), 6.70 (q, *J* = 1.2 Hz, 1H), 5.50-5.35 (m, 10H), 2.90-2.72 (m, 10H), 2.27 (q, *J* = 7.6 Hz, 2H), 2.16 (d, *J* = 1.2 Hz, 3H), 2.08 (quin, *J* = 7.6 Hz, 2H), 1.90 (quin, *J* = 7.6 Hz, 2H), 0.97 (t, *J* = 7.6 Hz, 3H); ^13^C NMR (100 MHz, CDCl_3_) δ 184.8, 183.6, 171.9, 149.3, 146.8, 136.9, 134.4, 133.8, 132.0, 129.4, 129.0, 128.9, 128.5, 128.3, 128.2, 128.1, 128.1, 127.8, 127.0, 125.0, 123.5, 33.6, 26.6, 25.6, 25.6, 25.6, 25.5, 24.3, 20.5, 16.1, 14.3; HRMS (ESI-LDI) calcd for C_31_H_36_O_4_Na ([M+Na]^+^) 495.2506, found 495.2505.

### Synthesis and characterization of 5-O-docosahexaenoyloxy-2-methyl-1,4-naphthoquinone (C22:6-Acyl plumbagin, 2j)

Following the general procedure, docosahexaenoyl chloride was prepared by treatment of docosahexaenoic acid (210 mg, 0.64 mmol) with oxalyl chloride (200 µL, 2.33 mmol) for 3 h. The reaction of plumbagin (**1**) (20 mg, 0.11 mmol) with the crude docosahexaenoyl chloride using DMAP (1.3 mg, 0.01 mmol) in pyridine for 3 h gave the crude product. The crude product was purified by silica gel column chromatography (EtOAc/hexanes = 1/20, v/v) to give **2j** (39 mg, 70%) as yellow oil; IR (neat) 3012, 2964, 2927, 2875, 1768, 1662, 1595, 1358, 1271, 1190, 1128, 1026, 980, 897, 783, 714 cm^−1^; ^1^H NMR (400 MHz, CDCl_3_) δ 8.06 (dd, *J* = 8.0 Hz, 1.2 Hz, 1H), 7.73 (t, *J* = 8.0 Hz, 1H), 7.35 (dd, *J* = 8.0 Hz, 1.2 Hz, 1H), 6.71 (q, *J* = 1.2 Hz, 1H), 5.34 (m, 12H), 2.92-2.73 (m, 12H), 2.60 (q, *J* = 7.2 Hz, 2H), 2.16 (d, *J* = 1.2 Hz, 3H), 2.07 (quin, *J* = 7.2 Hz, 2H), 0.97 (t, *J* = 0.4 Hz, 3H); ^13^C NMR (100 MHz, CDCl_3_) δ 184.8, 183.6, 171.5, 149.3, 146.8, 136.9, 134.4, 133.8, 132.0, 129.5, 129.4, 128.5, 128.2 (2C), 128.2, 128.1, 128.1, 128.1, 127.8, 127.8, 127.0, 125.1, 123.5, 34.1, 25.6, 25.6, 25.6, 25.6, 25.5, 22.4, 20.5, 16.1, 14.3; HRMS (ESI-LDI) calcd for C_33_H_38_O_4_Na ([M+Na]^+^) 521.2662, found 521.2661.

### Stability of C18:1-acyl plumbagin (2f) and C18:1-acyl juglone (5f) under basic conditions

A 2.2×10^−2^ M solution of Triton B in MeOH was added to a 2.2×10^−2^ M solution of each sample in 1,4-dioxane. Then, the mixture was diluted with 1,4-dioxane to a concentration of 1.1×10^−3^ M. Then, the stability of each compound was monitored by UV-vis spectroscopy. UV-vis spectra were measured at 25°C on a UV-vis spectrophotometer (JASCO V-650, Tokyo, Japan). For comparison, UV-vis spectra of 1.1×10^−3^ M solution of C18:1-acyl plumbagin (**2f**), and C18:1-acyl juglone (**5f**) in 1,4-dioxane and MeOH (19/1, v/v) were measured.

### Statistical analysis

All data are expressed as the mean value ± the standard deviation (SD) of at least three independent determinations for each experiment. Statistical significance between each experimental group was analyzed using Student's t-test, and a probability level of 0.01 and 0.05 was used as the criterion of significance.

## Results and Discussion

### Synthesis of 5-O-acyl plumbagins (2a–j)

**Figure 2:**
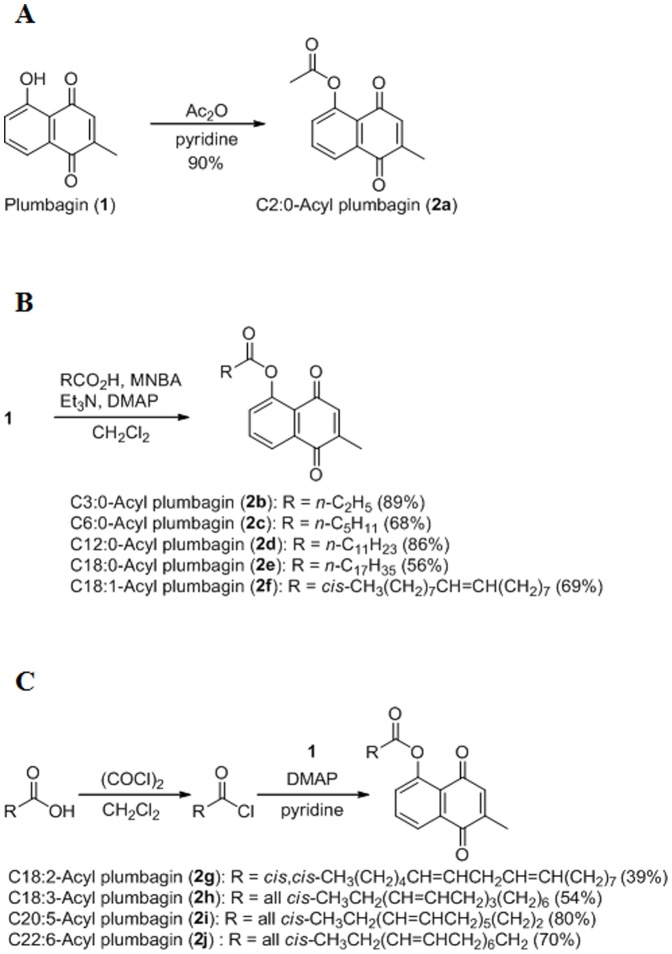
Synthesis of 5-*O*-acyl plumbagins (2a–j). (A) Synthesis of 5-*O*-acyl plumbagin (**2a**). (B) Synthesis of 5-*O*-acyl plumbagins (**2b–f**). (C) Synthesis of 5-*O*-acyl plumbagins (**2g–j**).

Synthesis of 5-*O*-acyl plumbagins (**2a–j**) is summarized in Fig. 2. C2:0-Acyl plumbagin (**2a**) was prepared by treatment of plumbagin (**1**) with acetic anhydride in pyridine in 90% yield (Fig. 2A). 5-*O*-Acyl plumbagins **2b–f** were prepared by condensation of plumbagin (**1**) with the corresponding carboxylic acids, using MNBA with triethylamine and DMAP in CH_2_Cl_2_ (Fig. 2B). 5-*O*-Acyl plumbagins **2g–j** were prepared by acylation of plumbagin (**1**) with acyl chlorides in the presence of DMAP in pyridine (Fig. 2C).

### Stability of C18:1-acyl plumbagin (2f) and C18:1-acyl juglone (5f) under basic conditions

**Figure 3:**
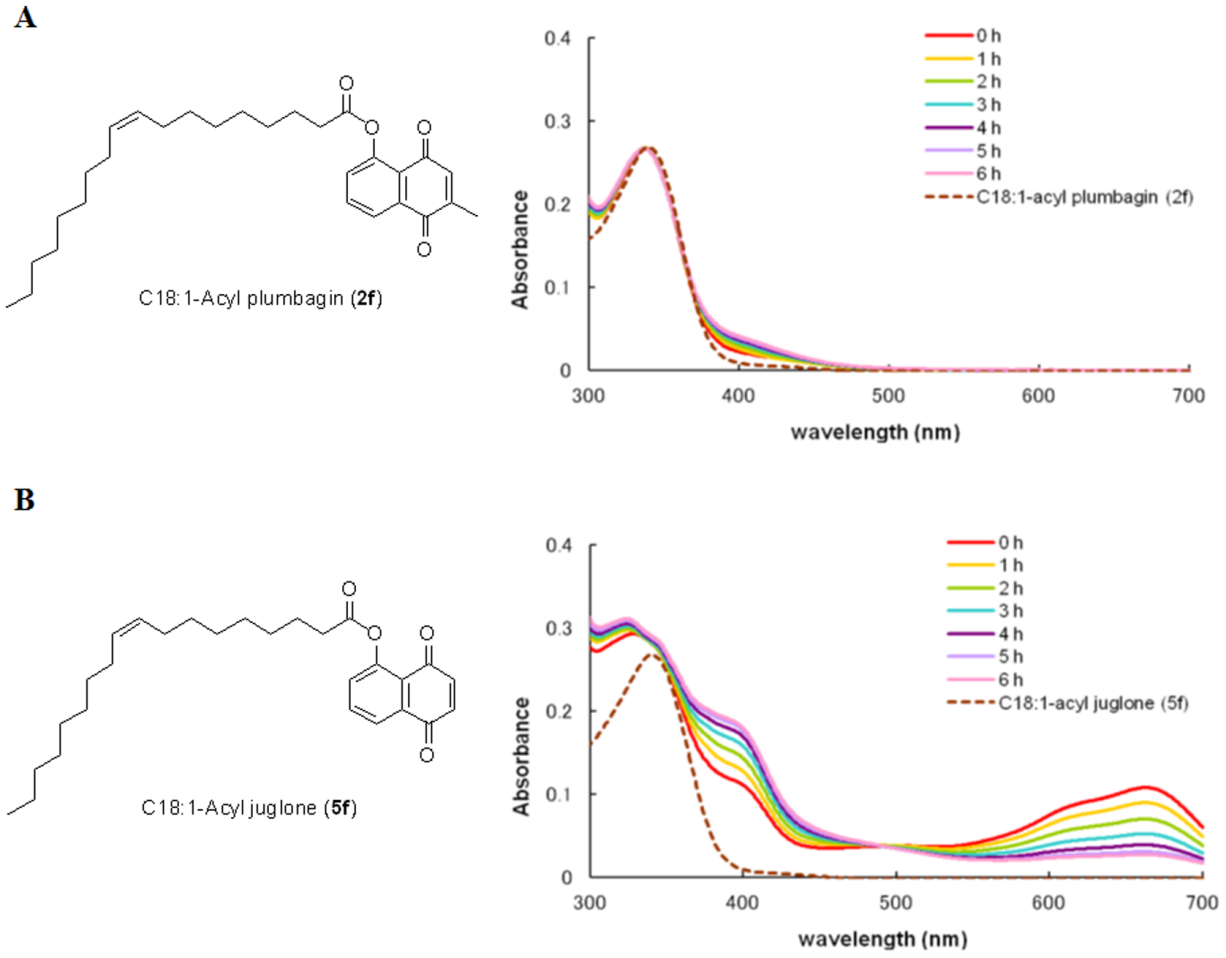
Stability of C18:1-acyl plumbagin (2f) and C18:1-acyl juglone (5f) under basic conditions. C18:1-acyl plumbagin (**2f**) (A) and C18:1-acyl juglone (**5f**) (B) were treated with 1 equivalent of Triton B in 1,4-dioxane and MeOH. The mixtures were monitored by UV-vis spectroscopy over time. Conditions: 1.1×10^−3^ M, 25°C, light path length = 1 mm.

We have found that C18:1-acyl plumbagin (**2f**) is more stable than C18:1-acyl juglone (**5f**) [23] under basic conditions. C18:1-acyl plumbagin (**2f**) and C18:1-acyl juglone (**5f**) were treated with Triton B (benzyltrimethylammonium hydroxide) in 1,4-dioxane and MeOH. As shown in Fig. 3, UV spectra of the mixture were recorded at different reaction times. Almost no changes in UV spectra of C18:1-acyl plumbagin (**2f**) were observed before and after treatment of Triton B (Fig. 3A). UV spectra of the mixture of C18:1-acyl plumbagin (**2f**) and Triton B rarely changed during the reaction. In contrast, the changes in UV absorption of C18:1-acyl juglone (**5f**) were clearly observed (Fig. 3B). UV absorption at 675 nm and 400 nm was observed soon after the addition of Triton B. The absorption at 675 nm suggests the formation of an extended aromatic compound by the reaction of C18:1-acyl juglone with Triton B in 1,4-dioxane and MeOH. The absorption at 675 nm became smaller during the reaction, suggesting that the extended aromatic compound had decomposed. The absorption at 400 nm increased with reaction time. We observed weak and broad absorption in the wavelength range 300–700 nm. These results suggest that C18:1-acyl juglone (**5f**) readily decomposes under basic conditions.

### Effect of synthesized 5-O-acyl plumbagins (2a–j) on the activities of mammalian pols

**Figure 4:**
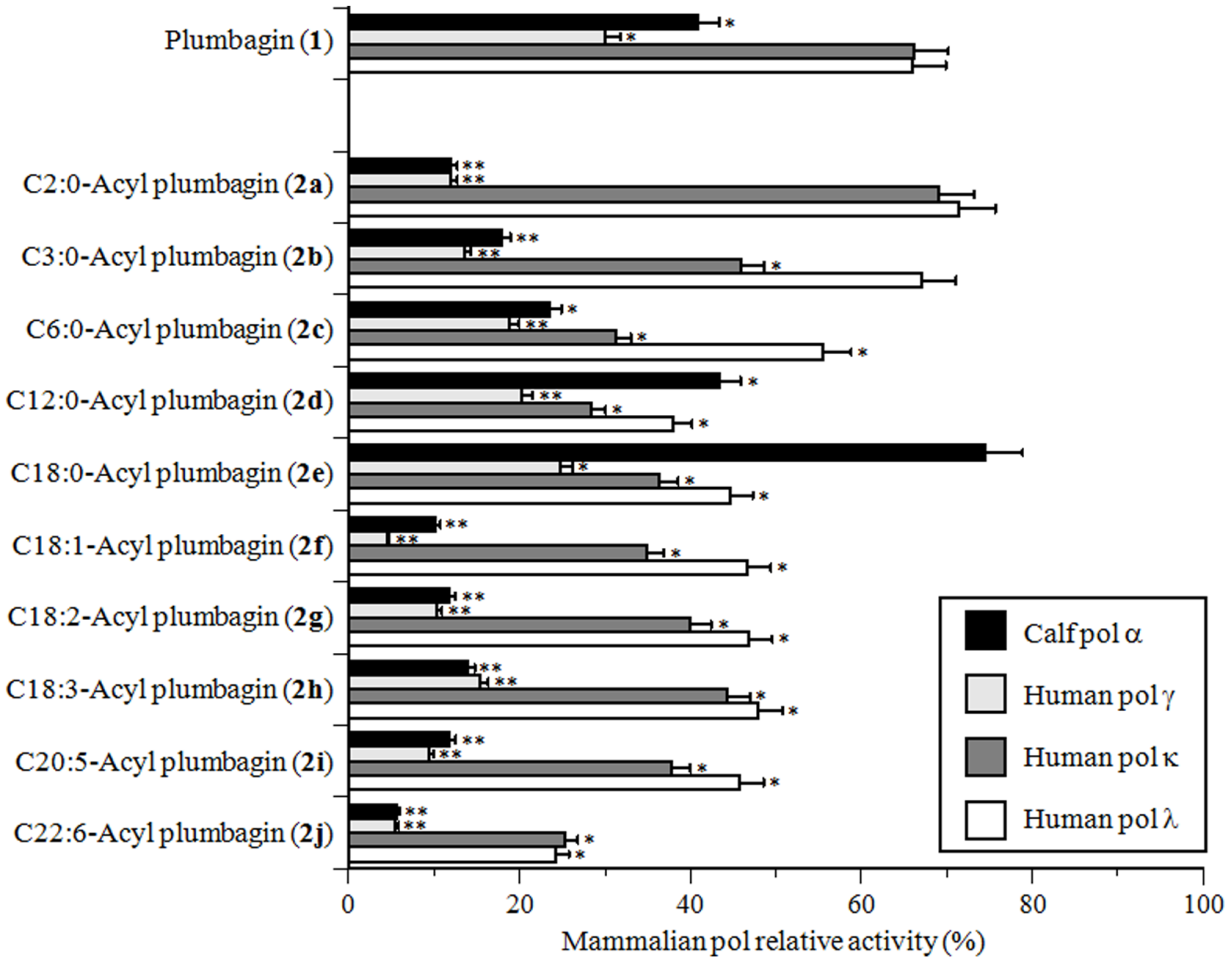
Inhibitory effects of plumbagin (1) and 5-*O*-acyl plumbagins (2a–j) on the activity of mammalian pols. Each compound (10 µM) was incubated with calf pol α (B-family pol), human pol γ (A-family pol), human pol κ (Y-family pol), and human pol λ (X-family pol) (0.05 units each). Pol activity in the absence of the compound (control) was taken as 100%, and the relative activity is shown. Data are shown as the mean ± SD (n = 3). ** *P*<0.01 and * *P*<0.05 vs. controls.

Initially, the inhibitory activity of each 5-*O*-acyl plumbagin (**2a–j**) toward mammalian pols was investigated using calf pol α, and human pols γ, κ, and λ. In mammalian pols, pols α, γ, κ, and λ were used as the representative pols for families B, A, Y, and X, respectively [8], [9]. Assessment of the relative activity of each pol at a set concentration (10 µM) of plumbagin (**1**) and the ten chemically synthesized compounds showed that some 5-*O*-acyl plumbagins were stronger inhibitors of these four mammalian pols than plumbagin (**1**) (Fig. 4). The plumbagins conjugated with C18 and longer chain unsaturated fatty acids (i.e., C18:1 to C22:6-acyl plumbagins **2f–j**) strongly inhibited the activities of pols α and γ, suggesting that the group of unsaturated longer acyl side chains might be an important structural characteristic of 5-*O*-acyl plumbagin for pol inhibition. C22:6-acyl plumbagin (**2j**) was the strongest inhibitor of human pols κ and λ among the compounds tested. In contrast, C2:0-acyl plumbagin (**2a**) hardly inhibited the activities of human pols κ and λ, and C18:0-acyl plumbagin (**2e**) did not inhibit pol α activity. When activated DNA (bovine deoxyribonuclease I-treated DNA) was used as the DNA template-primer substrate instead of synthesized DNA [poly(dA)/oligo(dT)_18_ (A/T = 2/1)] and dNTP was used as the nucleotide substrate instead of dTTP, the inhibitory effects of these compounds did not differ (data not shown).

### Effect of synthesized 5-O-acyl plumbagins (2a–j) on cultured human cancer cells

**Figure 5:**
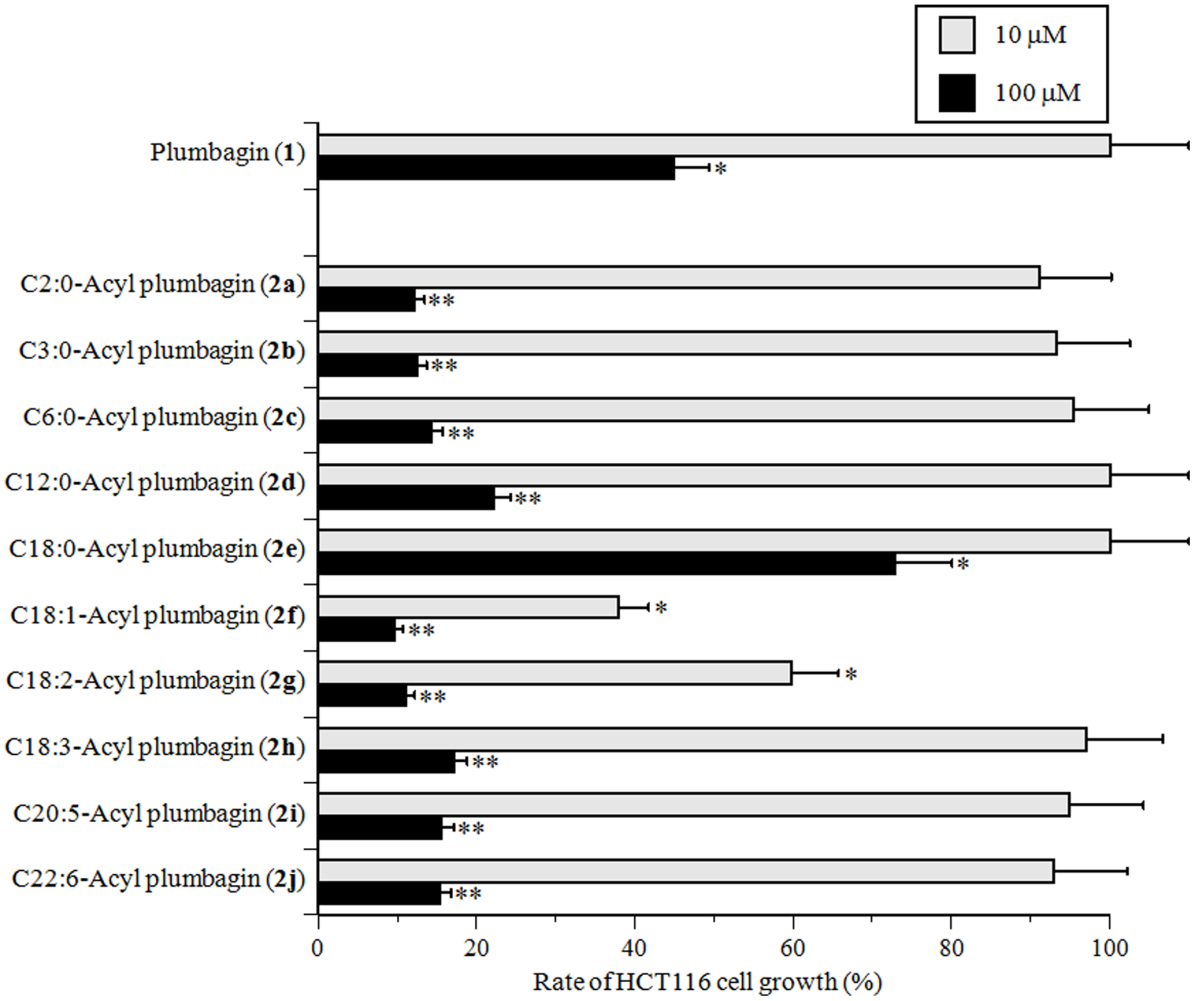
Effect of plumbagin (1) and 5-*O*-acyl plumbagins (2a–j) on the proliferation of HCT116 human colon carcinoma cells. Each compound (10 and 100 µM) was added to cultured HCT116 cells. The cells were incubated for 48 h, and the rate of proliferation inhibition was determined by MTT assay. Cell proliferation inhibition of the cancer cells in the absence of the compound (control) was taken as 100%. Data are shown as the mean ± SD (n = 5). ** *P*<0.01 and * *P*<0.05 vs. controls.

Pols have emerged as important cellular targets for chemical intervention in the development of anti-cancer agents [2]. Therefore, the synthesized 5-*O*-acyl plumbagins (**2a–j**) could be useful in chemotherapy. Hence, we investigated the cytotoxic effect of these compounds against HCT116 human colon carcinoma cells. As shown in Fig. 5, 100 µM plumbagin (**1**) approximately 50% suppressed cell proliferation, but 10 µM of this compound did not. Of the ten synthesized compounds, the nine 5-*O*-acyl plumbagins except for C18:0-acyl plumbagin (**2e**) were stronger cell proliferation inhibitors than plumbagin (**1**). C18:1-Acyl plumbagin (**2f**) and C18:2-acyl plumbagin (**2g**) had the first and second strongest proliferation inhibitory effect on HCT116 cells among the compounds tested, and at 10 µM compound these cells showed less than 40 and 60% of the cell proliferation rate, respectively.

### Relationship of mammalian pol inhibition and human cancer cell proliferation suppression by 5-O-acyl plumbagins (2a–j)

**Figure 6:**
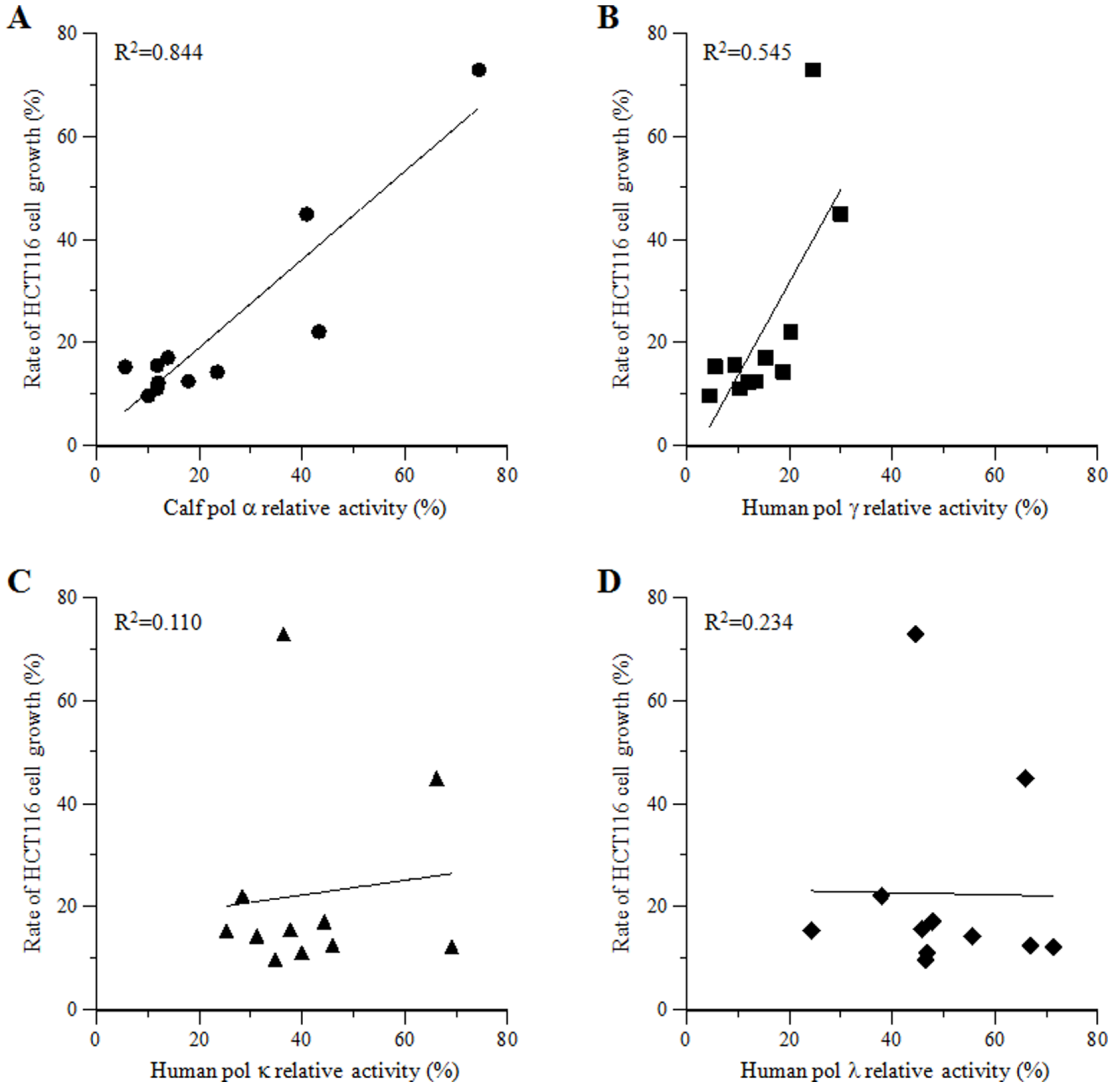
Relationship between mammalian pol inhibitory activities versus human cancer cell proliferation inhibition by plumbagin (1) and 5-*O*-acyl plumbagins (2a–j). X-axis indicates mammalian pol relative activity at 10 µM compound. (A) Calf pol α, (B) human pol γ, (C) human pol κ, and (D) human pol λ. Y-axis indicates rate of HCT116 human colon carcinoma cell proliferation at 100 µM compound. These data are based on Figs. 4 and 5; correlation coefficient values are shown in each panel.

The possible relationship between the observed inhibition of four mammalian pol families and HCT116 human colon cancer cell proliferation inhibitory activity was confirmed by comparing the effects of plumbagin (**1**) and the ten synthesized 5-*O*-acyl plumbagins (**2a–j**) on these biological activities (Fig. 6). The effect of 10 µM of these compounds on the relative activity of pol α, which is a DNA replicative pol of the B family, showed the highest correlation with the effect of 100 µM of these compounds on the cancer cell proliferation among those mammalian pol families tested, with a correlation coefficient of 0.844. The relative activity of pol γ, a mitochondrial DNA replicative pol of the A family, showed a moderate correlation (R^2^ = 0.545) with the cancer cell proliferation rate. Conversely, neither the activities of pols κ and λ, which are DNA repair-related pols of the Y and X families respectively, were related to cytotoxicity, with a correlation coefficient between these activities and cytotoxicity of <0.25. These results led to the speculation that the inhibition of the activities of DNA replicative pols, such as pols α and γ; in particular, inhibition of both the A and B families of pols by compounds **1** and **2a–j** might cause the suppression of human cancer cell proliferation.

In particular, C18:1-acyl plumbagin (**2f**) showed the strongest inhibition of pol γ of the ten synthesized 5-*O*-acyl plumbagins (**2a–j**) tested. This compound also exhibited the strongest effects on cancer cell proliferation suppression (Fig. 5). Therefore, C18:1-acyl plumbagin (**2f**) was used in the latter part of this study.

### Effects of C18:1-acyl plumbagin (2f) on the activities of various pols and other DNA metabolic enzymes

As described briefly in the Introduction, we succeeded in obtaining eleven mammalian pol species, including pols α, β, γ, δ, ε, η, ι, κ, λ, and μ, and TdT; however, pols ζ, θ and ν, and REV1 were not yet available at the time of the study. Currently, eukaryotes are thought to express at least 15 species of pols [6], [7], and we are still in an era when most pols are very difficult to obtain in their purified form in a laboratory. Table 1 shows the inhibitory effect (50% inhibitory concentration; IC_50_ value) of C18:1-acyl plumbagin (**2f**) against various pol species including the 11 mammalian pols that could be obtained. This compound inhibited the activity of all of the mammalian pols with IC_50_ values of 4.5–10.6 µM, and 50% inhibition of the A, B, X, and Y families of pols was observed at doses of 4.5, 4.8–5.3, 9.2–10.6, and 7.6–8.2 µM, respectively; therefore, the inhibitory effect of this compound on the A and B families of pols was 1.5- to 2-fold stronger than that on the X and Y families of pols. Because the IC_50_ values of aphidicolin, a known eukaryotic DNA replicative pol α, δ, and ε inhibitor, were 20, 13, and 16 µM, respectively [45], the pol inhibitory activity of C18:1-acyl plumbagin (**2f**) was >2-fold more potent than that of aphidicolin.

**Table 1:** IC_50_ of C18:1-acyl plumbagin (**2f**) on the activities of mammalian pols, other species pols, and various DNA metabolic enzymes.

Enzyme	IC_50_ values (µM)
– *Mammalian pols* –	
[A family of pol]	
Human pol γ	4.5±0.40 **
[B family of pols]	
Calf pol α	4.8±0.38 **
Human pol δ	5.3±0.41 **
Human pol ε	4.9±0.40 **
[X family of pols]	
Rat pol β	10.3±0.46 **
Human pol λ	9.2±0.43 **
Human pol μ	9.5±0.48 **
Calf TdT	10.6±0.50 **
[Y family of pols]	
Human pol η	7.9±0.42 **
Mouse pol ι	8.2±0.43 **
Human pol κ	7.6±0.41 **
– *Plant pol* –	
Cauliflower pol α	>100
Rice pol λ	>100
– *Prokaryotic pols* –	
*E. coli* pol I	>100
*Taq* pol	>100
T4 pol	>100
– *Other DNA metabolic enzymes* –	
Calf primase of pol α	>100
T7 RNA polymerase	>100
Mouse IMP dehydrogenase (type II)	>100
T4 polynucleotide kinase	>100
Bovine deoxyribonuclease I	>100

Compounds were incubated with each enzyme (0.05 units). Enzyme activity in the absence of an inhibitor (control) was taken as 100%; data, mean ± SD (n = 3).

** *P*<0.01 vs. controls.

In contrast, C18:1-acyl plumbagin (**2f**) had no effect on plant pols such as cauliflower pol α or rice pol λ, or prokaryotic pols, such as *E. coli* pol I, *Taq* pol, or T4 pol (Table 1). The three-dimensional structures of eukaryotic pols are likely to differ greatly from those of prokaryotic pols. This compound did not inhibit the activity of other DNA metabolic enzymes, such as calf primase pol α, 7 RNA polymerase, mouse IMP dehydrogenase (type II), T4 polynucleotide kinase, or bovine deoxyribonuclease I. These results suggest that 5-*O*-acyl plumbagins (**2**) may be selective inhibitors of mammalian pols; in particular, plumbagin (**1**) conjugated with unsaturated fatty acids, such as C18:1-acyl plumbagin (**2f**), potently inhibited the activities of the A and B families of pols.

### Influence of C18:1-acyl plumbagin (2f) on the hyperchromicity of dsDNA

**Figure 7:**
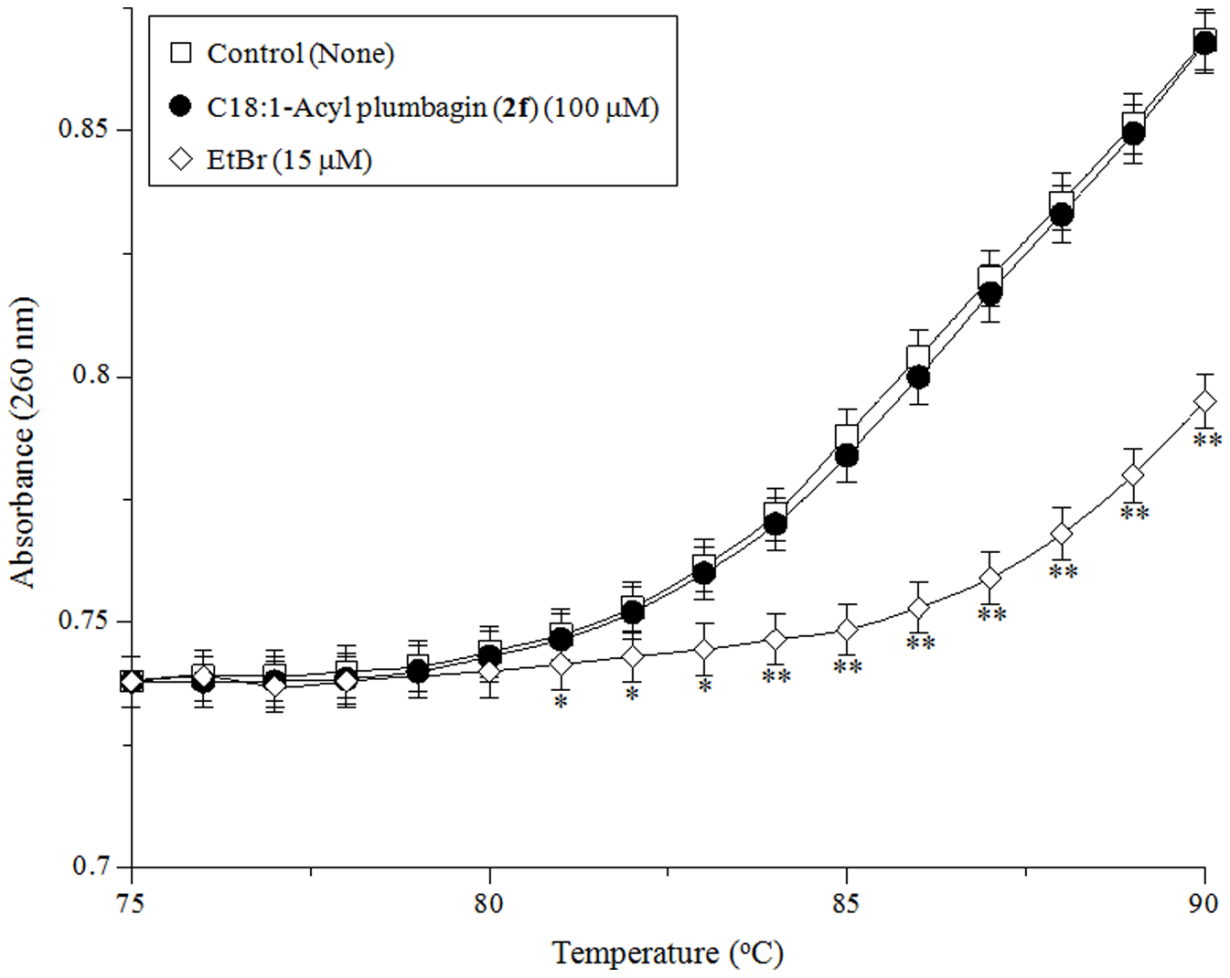
Effect of C18:1-acyl plumbagin (2f) on the thermal transition of dsDNA. Control (open-square), C18:1-acyl plumbagin (**2f**) (100 µM, closed-circle), and EtBr (15 µM, open-diamond) were incubated with 6 µg/mL of calf thymus dsDNA in 0.1 M Na-phosphate buffer (pH 7.0). Data are shown as the mean ± SD (n = 3). ** *P*<0.01 and * *P*<0.05 vs. controls.

Specific assays were performed to determine whether C18:1-acyl plumbagin (**2f**)-induced inhibition resulted from the ability of the compound to bind to DNA or the enzyme. The interaction of C18:1-acyl plumbagin (**2f**) with dsDNA was investigated by studying its thermal transition. For this, the melting temperature (T_m_) of dsDNA in the presence of an excess of C18:1-acyl plumbagin (**2f**) (100 µM) was observed using a spectrophotometer equipped with a thermoelectric cell holder. As shown in Fig. 7, a thermal transition (i.e., T_m_) from 75 to 90°C was not observed within the concentration range used in the assay, whereas when a typical intercalating compound, such as ethidium bromide (EtBr, 15 µM), was used as a positive control, an obvious thermal transition was observed.

The question of whether the inhibitory effect of C18:1-acyl plumbagin (**2f**) resulted from nonspecific adhesion to mammalian pols or from its binding to these enzymes was investigated by determining if an excessive amount of nucleic acid [poly(rC)] or protein (BSA; bovine serum albumin) prevented the inhibitory effect of C18:1-acyl plumbagin (**2f**). Poly(rC) and BSA had little or no influence on pol inhibition by C18:1-acyl plumbagin (**2f**) (data not shown), suggesting that this compound selectively bound to the pol molecule. These observations indicated that C18:1-acyl plumbagin (**2f**) did not act as a DNA intercalating agent or as a template-primer substrate.

Collectively, these results suggested that C18:1-acyl plumbagin (**2f**) might be a potent and specific inhibitor of mammalian pols. Subsequently, we investigated whether pol inhibition by C18:1-acyl plumbagin (**2f**) resulted in reduced human cancer cell proliferation.

### Effect of C18:1-acyl plumbagin (2f) on cultured human cancer cell lines

C18:1-Acyl plumbagin (**2f**) treatment for 48 h suppressed the proliferation of various human cancer cells in a dose-dependent manner. As shown in Table 2, C18:1-acyl plumbagin (**2f**) prevented the proliferation of nine human cancer cell lines, such as A549, DU145, HCT116, HeLa, HepG2, HT-29, MCF-7, PANC-1, and PC3 cells, with LD_50_ values of 2.9–21.0 µM. These results suggested that this compound could have suppressive activity against the different type of cancer cell lines. In particular, this compound showed the strongest cell proliferation suppression in the colon cancer cell lines, HCT116 and HT-29, with LD_50_ values of 6.5 and 2.9 µM, respectively. These dose-response curves by MTT detection were the same as that obtained by trypan blue staining (data not shown), suggesting that C18:1-acyl plumbagin (**2f**) might cause a direct toxic effect on the cells. Because these LD_50_s were similar to the IC_50_s for pols (Table 1), this inhibition must be mostly led by the function of pols, such as DNA replicative pol α. C18:1-Acyl plumbagin (**2f**) more strongly suppressed the proliferation of these human cancer cell lines than aphidicolin, which is an inhibitor of eukaryotic DNA replicative pols (data not shown).

**Table 2:** LD_50_ values of C18:1-acyl plumbagin (**2f**) on the proliferation of human cancer cells.

Human cancer cell line	LD_50_ values (µM)
A549 (lung cancer)	15.4±1.7 **
DU145 (prostate cancer)	8.8±0.9 **
HCT116 (colon cancer)	6.5±0.6 **
HeLa (cervical cancer)	21.0±2.5 **
HepG2 (hepatocellular liver cancer)	7.4±0.8 **
HT-29 cells (colon cancer)	2.9±0.4 **
MCF-7 (breast cancer)	7.2±0.7 **
PANC-1 (pancreatic cancer)	16.1±1.8 **
PC3 (prostate cancer)	10.0±1.1 **

The nine human cancer cell lines were incubated with C18:1-acyl plumbagin (**2f**) for 48 h. Cell viability was determined by MTT assay, and this viability in the absence of an inhibitor (control) was taken as 100%; data, mean ± SD (n = 5).

** *P*<0.01 vs. controls.

### Effect of C18:1-acyl plumbagin (2f) on *in vivo* anti-tumor activity

Because the cell proliferation inhibitory effect of C18:1-acyl plumbagin (**2f**) was the strongest on HT-29 cells among the nine human cancer cell lines tested (Table 2), this cell line was used *in vivo* anti-tumor assays. In this assay, we investigated whether C18:1-acyl plumbagin (**2f**) was more stable and/or had stronger bioactivity than C18:1-acyl juglone (**5f**) *in vivo*; thus, C18:1-acyl plumbagin (**2f**) was compared with vitamin K_3_ (**3**), juglone (**4**), and C18:1-acyl juglone (**5f**).

**Figure 8:**
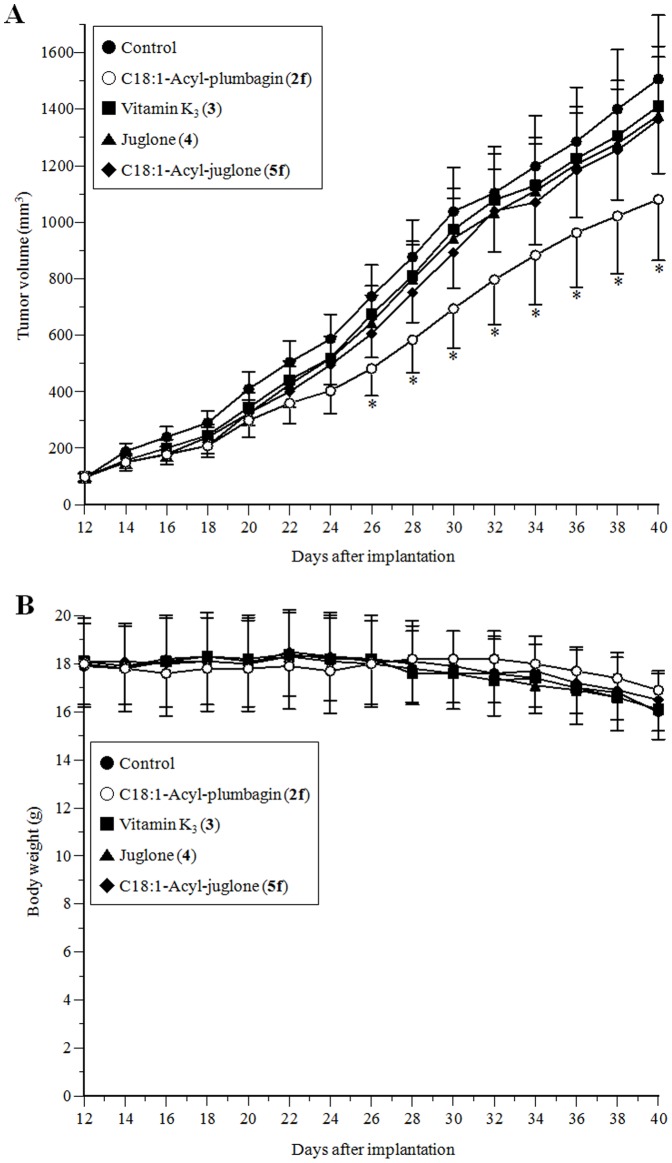
*In vivo* anti-tumor effects of C18:1-acyl plumbagin (2f). Nude mice bearing HT-29 solid tumors were administered with PBS (control), vitamin K_3_ (**3**), juglone (**4**), C18:1-acyl juglone (**5f**), and C18:1-acyl plumbagin (**2f**) at a dose of 5 mg/kg. (A) Inhibitory effect on tumor volume in nude mice. (B) Body weight changes of nude mice; data, means ± SE (n = 6). * *P*<0.05 vs. controls.

HT-29 cells were subcutaneously injected into 40 nude mice. At 12 days after the implantation, these nude mice were sorted five groups (one control group and four treatment groups) and each mouse group contained 6 mice bearing solid tumor volume of 96–102 mm^3^. The sorted nude mice were injected with each test compound dissolved in PBS (5 mg/kg) at 1-day intervals until 40 days. As shown in Fig. 8A, these compounds suppressed tumor growth as compared with the control (PBS) group, and the decreased rates of tumor volume at 40 days following injection with vitamin K_3_ (**3**), juglone (**4**), C18:1-acyl juglone (**5f**), and C18:1-acyl plumbagin (**2f**) were 7.0, 9.2, 10.3, and 30.5%, respectively. C18:1-acyl plumbagin (**2f**) showed more than a 3-fold stronger anti-tumor effect than the other compounds tested, suggesting that C18:1-acyl plumbagin (**2f**) must be stable *in vivo*, but C18:1-acyl juglone (**5f**) did not. A significant correlation was found between 5-*O*-acyl plumbagins (**2**) and the inhibition of mammalian pols, especially DNA replicative pol α (Fig. 6A). C18:1-Acyl plumbagin (**2f**) may be able to penetrate cancer cells of tumor in mouse and reach the nucleus, inhibiting the activities of pols and then the inhibition of pol activity by this compound may lead to cell proliferative suppression and prevent tumor growth.

None of the nude mice showed any significant loss of body weight throughout the experimental period (Fig. 8B). It was also noted that the main visceral organs, such as the liver, lung, kidney, spleen, heart, stomach, small intestine, large intestine, pancreas, and testis of all the groups showed no toxic or degenerative histological appearance (data not shown); therefore, C18:1-acyl plumbagin (**2f**) is of interest as a candidate material for anti-cancer treatment.

## Conclusions

We previously found that vitamin K_3_ (2-methyl-1,4-naphthoquinone), juglone (5-hydroxy-1,4-naphthoquinone) conjugated with fatty acids inhibited the activity of mammalian pols. In this study, 5-*O*-acyl plumbagins (**2**), which are plumbagins (5-hydroxy-2-methyl-1,4-naphthoquinone) conjugated with fatty acid, were produced to establish an efficient chemical synthesis method. In the synthesized ten 5-*O*-acyl plumbagins (**2a–j**), **2c–j** are novel compounds. These synthesized compounds were stronger inhibitors of the mammalian pols α, γ, κ, and λ representing the pol families B, A, Y, and X, respectively, than plumbagin (**1**). Of the synthesized compounds, C18:1-acyl plumbagin (**2f**) showed the strongest suppression of human cancer cell proliferation. The human cancer cytotoxicity of this compound was realized through the inhibition of pols, which are essential for DNA replication as well as cell division. Because C18:1-acyl plumbagin (**2f**) potently inhibited the activities of replicative pols, such as pols α and γ and suppressed human cancer cell proliferation, they might show *in vivo* anti-tumor activity without any side effects. The *in vivo* anti-tumor effect of C18:1-acyl plumbagin (**2f**) was stronger than that of C18:1-acyl juglone (**5f**), because C18:1-acyl plumbagin (**2f**) is more stable than C18:1-acyl juglone (**5f**) under basic conditions. These results suggested that 5-*O*-acyl plumbagins (**2**), such as C18:1-acyl plumbagin (**2f**), could be used as anti-cancer chemotherapy agents based on their mammalian pol inhibition.
